# 

**Published:** 1885-10-20

**Authors:** 


### TABLE OF CONTENTS.

ORIGINAL AND SELECTED ARTICLES:

An Interesting Case Involving the Reputation of a Medical Brother, by P. S. Verdery, M D., of Douglassville, Ga , 361. Convallaria Majalis, by G. M. Garland, M.D., Assistant in Clinical Medicine, Harvard University, Boston, Mass., 366. The Treatment of Vomiting with Large Doses of Oxalate of Cerium, by W. J Chittick, M. D , 368. Plaster of Paris in the Treatment of Fractures: Report on Surgery from the First Congressional District to the Medical Association of Georgia, at its Thirty-sixth Annual Session at Savannah, April, 1885, by W. H Elliot, M D., Chairman. Savannah, Ga., 372. Oleate of Mercury in Obstinate Hepatic Congestions, by C. A. Bryce, M.D., of Richmond, Va., 374. Bacteria as Therapeutic Agents, 375. Nasal Catarrh, 376. Psoriasis, 377.

ABSTRACTS AND GLEANINGS:

Hemorrhagic Malarial Fever, 378. When Quinine fails, What then? 481. A Case of Cholera Infantum, Treated with Large Doses of Morphine and Atropia, 383. Scutellaria Lateriflora in the Tre tment of Enuresis, 384. Treatment for Malaria; A Valuable Remedy for Headache, 385 Never Overlook an Over-distended Bladder, 386. Hydrobromate of Quinine and Valerinate of Caffeine in the Treatment of Malarial Poisoning; A Novel Cure for Rheumatism, 387. Local Anæsthetics; Morphine in Cholera Infantum, 388. Coincidental Variola and Vaccinia; An Improvement in the Treatment of Nævus by Sodium Ethylate, 389. The Uses of Cocaine; Extirpation of the Lung, 390.

SCIENTIFIC ITEMS:

Is the Air Colorless; Forcite Powder, 391. The Minuteness of Germs; Indian-Ink, 392.

PRACTICAL NOTES AND FORMULÆ.

Ointment for Hæmorrhoids; For Asmatic Paryoxysm; Laxative Syrup; For Children's Dysentery, 393. Bengal Cholera Pill; Huchird's Anti-Tubercular Pills; Laxative Sugar; Application to the Gums in Dentition; Toothache Drops; Bright's Disease, 394.

EDITORIALS AND MISCELLANEOUS.

Notices; John C. Barker & Co.'s Cod Liver Oil; A New National Association, 395; Fairchild, Bro. & Foster; Morphine for Quinine; The International Congress will be Held in the United States; Doctors in Atlanta; International Medical Congress, 397. Books and Pamphlets Received, 398.

